# Acute Generalized Peritonitis in a Peripheral Hospital Centre in Benin: Can It Be Managed by a Local General Practitioner?

**DOI:** 10.1155/2021/5543869

**Published:** 2021-04-19

**Authors:** Semevo Romaric Tobome, Adrien Montcho Hodonou, Anifa Wahide, Kadiri Alassan Boukari, Moïse Kponou, Christelle Hermione Elvire Bankole, Roberto Caronna

**Affiliations:** ^1^Atacora Departmental Hospital Center, Atacora, Benin; ^2^General Surgery Department, Centre Hospitalier Départemental Universitaire de Parakou, Parakou, Benin; ^3^Centre de Santé Communal de Natitingou, Zone Office Natitingou, Boukoumbé Toucountouna, Natitingou, Benin; ^4^Department of Surgical Sciences, Sapienza University of Rome, Rome, Italy

## Abstract

**Background:**

Acute generalized peritonitis in resource-poor countries is still a health challenge due to late diagnosis, surgical delay, and specialists' unavailability. These are the foremost determinants of surgical morbidity and mortality. We report the experience of a peripheral hospital in Benin not equipped with specialized surgeons.

**Methods:**

This is an observational, retrospective, and descriptive study including patients operated for acute generalized peritonitis at the Atacora Departmental Hospital Centre, Benin, where unfortunately CT scan and intensive care unit are still not available. Most of surgical activities were performed by a general practitioner with previous surgical training (but no surgical specialization). Age, gender, cause of peritonitis, surgical procedures, and postoperative outcome were evaluated.

**Results:**

Sixty-three patients were included. The mean age was 23.2 years and sex ratio M/F 1.5. The mean surgical delay was 26 hours (range: 6–92 hours). An ileal typhoid perforation was found in 40 patients (63.5%), and 35 of them (87.5%) underwent a primary perforation repair without bowel resection. 73% of surgical procedures were performed by the general practitioner. Morbidity was 34.9% and mortality was 14.3%. The average postoperative hospital stay was 12 days (range: 11–82 days). These results were comparable to those observed in the subgroup of patients (17 cases) operated by the general surgeons (morbidity 32.6%, mortality 13.0%, and average postoperative hospital stay 11 days, range: 1–58 days).

**Conclusion:**

Acute generalized peritonitis requires urgent management, and it can be effectively carried out, in a context of limited resources, by a general practitioner with surgical skills.

## 1. Introduction

Due to etiological heterogeneity, acute generalized peritonitis (AGP) remains a frequent abdominal emergency with high lethality. Several factors including patientʼs conditions, onset, cause, and quality/promptness of treatment significantly affect the outcome [1]. In sub-Saharan Africa, most patients look at modern medical care only after traditional treatment failure. Moreover, lack of financial resources entails delayed hospitalization until evident critical conditions and lack of specialized physicians make the surgical management late or unfeasible. Most of AGP in this African context are commonly caused by nontraumatic ileal perforations, often of typhoid origin due to the unavailability of clean water.

We report the results of our experience in a peripheral hospital centre in Benin (sub-Saharan Africa), addressing etiological and therapeutic aspects.

## 2. Materials and Methods

This study was carried out at the Centre Hospitalier Départemental de lʼAtacora (CHD-A), Benin. This is a referral hospital of the northern part of Western Benin (891,528 inhabitants) [2], placed in Natitingou, the Departmentʼs main town. Since July 2019, this hospital has undergone a redefinition of its activities becoming a general hospital. Unfortunately, CHD-A does not have the intensive care unit, CT scan, and microbiological laboratory.

This is an observational, retrospective, and descriptive study, with a data collection from March 1^st^ 2018 to November 30^th^ 2019 (21 months). Patients admitted to CHD-A for abdominal pain underwent clinical examination, standard abdominal radiography, and basic laboratory exams (blood count and blood typing).

All patients received a preoperative medical approach, essentially involving hydroelectrolytic imbalance correction, hemodynamic stabilization, empirical broad-spectrum antibiotics infusion, and analgesics.

Patients who died before laparotomy were excluded from this study, while all patients admitted for abdominal pain due to AGP confirmed at laparotomy were included. Age, gender, surgical delay (time between admission to CHD-A and laparotomic incision), ASA score (American Society of Anesthesiologists Score) [3], cause of peritonitis disclosed at laparotomy, type of operation, postoperative outcome, and length of hospital stay were the variables considered. Patient's data were obtained from the surgical and anesthesiological reports. Depending on patients' clinical conditions, a locoregional anesthesia (spinal anesthesia supplemented by general anesthesia with mask) or a general anesthesia with orotracheal intubation was performed before laparotomy.

The majority of laparotomies were carried out by a permanent medical general practitioner graduated since three years, with surgical skills acquired by attending African and European hospitals, but not yet admitted to any surgical specialization. Two general surgeons were occasionally present.

Each patient discharged was systematically reviewed 7–10 days later, and all were monitored for 30 days after discharge. Postoperative complications were recorded according to the Clavien–Dindo classification [4].

Quantitative data were expressed as averages ± standard deviation and qualitative data as frequency and percentage. Overall survival (OS) was defined as the interval between surgery and last follow-up or death.

This study was approved by the Ethics Committee of Centre Hospitalier Départemental de lʼAtacora (CHD-A), Benin (Ref. N. 045/2020), and was conducted following the rules of the Helsinki Declaration of the World Medical Association.

## 3. Results

### 3.1. Sixty-Three Patients were Included and Operated for AGP

There were 38 males (60.3%) and 25 females (39.7%), with a sex ratio of 1.5. The mean age of the patients was 23.2 ± 17.7 years (range: 2–75 years). 75% of patients were less than 33 years old (Table 1).

Surgical delay was 26 hours (range: 6–92 hours). Nineteen patients (30.2%) were operated within 24 hours, and 32 (50.8%) were operated between 24 and 48 hours after admission.

On admission, the hemoglobin level ≤8 g/dL was detected in 22 patients (34.9%), and they received pre- or intraoperative blood transfusions. Most patients were in critical clinical conditions (ASA score ≥ 3), but sixteen (25.4%) were in extremely serious conditions (ASA score 4) (Table 1).

AGPs causes are presented in Table 2. There were 40 AGPs (63.5%) by nontraumatic ileal perforations (on the antimesenteric rim with enlarged mesenteric nodes) presumably of typhoid origin.

Anesthesiological and surgical procedures are given in Table 3.

Most laparotomies were performed by the general practitioner (46/63 cases, 73%), while the remaining 17 cases (27.0%) were operated by two general surgeons.

**Figure 1:**
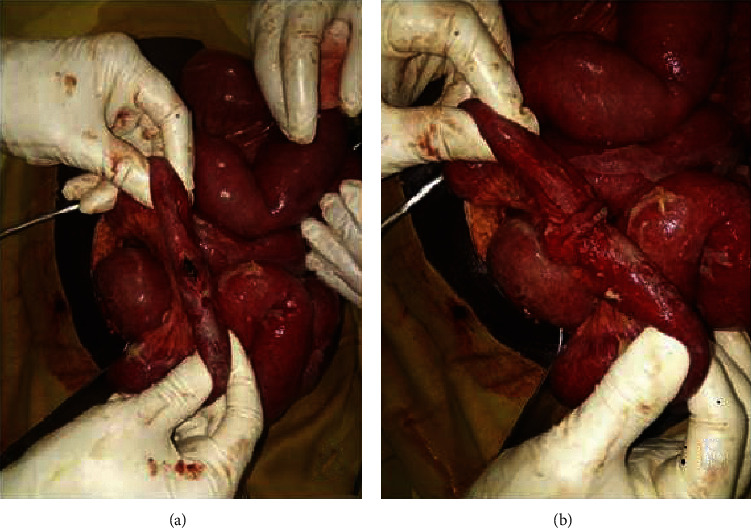
Typhoid ileal perforation (a) and primary repair (b). Primary repair was often adopted and consists of a single-layer suture with 2–4 large Vicryl 2/0 “U” stitches passed through the seromuscular intestinal layer far from perforation where less inflammatory involvement is evident, without edge excision and achieving a good introflection.

In patients with ileal perforation of presumed typhoid origin, a primary repair without resection/anastomosis was the main performed surgical procedure (55.6%) (Figure 1). All patients received a peritoneal lavage and peritoneal cavity drains: 3 drains in 34 patients (54.0%), 2 drains in 21 patients (33.3%), and 1 drain in 8 patients (12.7%). The average operating time was 61 ± 19 minutes (range 45–130 minutes), and the mean drains removal time was 6 ± 2 days (range 1–15 days).

Postoperative outcomes were unremarkable and uncomplicated in 41 patients (65.1%), but we observed several postoperative complications (morbidity 34.9%), more than one in few patients, as given in Table 4 according to Clavien–Dindo classification.

The average postoperative hospital stay was 12 days (range: 11 hours–82 days), and the overall mortality was 14.3%.

In the patients subgroup operated by the general surgeons (17/63 cases), morbidity was 32.6%, mortality was 13.0%, and the average postoperative hospital stay was 11 days with range of 1–58 days, comparable to those observed in the general practitioner treated group.

## 4. Discussion

Diagnosis of AGPs in poor settings is mainly based on physical examination with few diagnostic imaging and laboratory data. With remarkable frequency, AGPs are the most important abdominal surgical emergency at CHD-A, as confirmed by a Northern Benin District Hospital study [5]. The same came up from other sub-Saharan Africa countries, notably Togo, Burkina Faso, and Niger [6–8]. AGPs are often a consequence of the unfavorable evolution of typhoid infection not or badly managed. Primary and secondary prevention could be crucial to decrease AGPs incidence, and a greater commitment from the local authorities is still needed and waited.

Patients with AGP were frequently admitted to CHD-A in critical clinical conditions (ASA score 3 or 4) with a strong prognostic negative impact.

All ages were affected, but young patients (mean age: 23 years, range: 2–40) were the most affected (in our series 82.5%), as reported also by studies carried out in Burkina Faso [7], Togo [6], and the Central African Republic [9] (24, 25, and 23 years, respectively). All these studies confirmed male predominance [5–7, 9].

The most common cause of AGP was nontraumatic ileal perforation of presumed typhoid origin. Indeed, poor hygiene conditions and troubles of access to potable water, especially in rural areas, suggest typhoid origin. Moreover, salmonellosis qualified as a disease of “dirty hands” frequently affects in this context children and adolescents who are effectively the most represented in our series (patients <20 years old account for 40% of all individuals with ileal perforation). Several studies carried out both in Benin sub-regions and in other sub-Saharan countries report the same results (Table 5) [5–7] to confirm the evidence of this major public health problem [10, 11].

Elsewhere, as in Togo and Madagascar, gastric or duodenal ulcer perforations were the most frequent cause of nontraumatic AGP [12, 13], while in Central African Republic, appendicular AGPs were the most common [9]. Other causes, such as trauma, tuberculosis, Crohnʼs disease, and tumors, have been also reported by others [14–16] according to the context, environment, and lifestyle of each country.

Divergent views also exist about the best treatment of typhoid ileal perforation. Different surgical procedures have been reported, namely, primary repair, excision and suture, ileostomy only, resection, and ileoileal anastomosis. The choice may depend on various factors including cause of peritonitis, location and perforations' number, patient's conditions, and surgeon's experience.

Mehinto et al. (Cotonou) performed ileal resections followed by immediate terminal ileoileal anastomosis in 94.2% of cases with a morbidity of 21.2% [17]. Ouangre and Kambire (Burkina Faso) preferred ileostomy or excision followed by ileal suture, similar to Sambo (Benin) and Kassegne (Togo) [18].

In our experience, the average number of ileal perforations was 3, and a single perforation occurred in 47.5%. Primary ileal repair was the most common procedure (87.5%) at CHD-A (Figure 1). The attitude of the CHD-A operators towards ileal perforation primary repair is based on the results of a study carried out in Benin peripheral hospital in 2013. The objective of this study was to compare primary ileal suture with the ileal resection in patients with typhoid ileal perforations. The authors showed that primary ileal repair offered more advantages than ileal resection and anastomosis, especially in terms of morbidity [19].

Nonetheless, it is evident that AGP-related morbidity and mortality remains high. Sambo (Benin), Ouangre (Burkina Faso), and Kassegne (Togo) report morbidity of 39.6%, 40.2%, and 59.3%, respectively [5–7], higher than ours (34.9%).

Furthermore, mortality is still unfortunately very high. The rates reported by Doui Doumgba (Central African Republic) (7.5%), Sambo (Benin) (11.3%), Kassegne (Togo) (14.8%), and Ouangre (Burkina Faso) (19%) were, respectively, lower and higher than in our series (14.3%) [5–7, 9]. Patients' clinical conditions, prompt, or late management with perioperative “resuscitation” and successful surgical treatment are surely crucial for AGP prognosis.

Finally, few comments we would like to do about anesthesia. In AGP patients, anesthesia is frequently performed as general anesthesia with orotracheal intubation. However, almost half of our patients were operated under regional anesthesia (spinal anesthesia) with sedation and mask ventilation. This choice was motivated by the attempt to limit the surgical trauma. As described, AGP diagnosis was always performed clinically (advanced radiological imaging not available) often in critically compromised patients (ASA 3 and ASA 4). Therefore, laparotomy started with a median limited suprapubic/periumbilical incision under regional anesthesia and sedation. When the bowel perforation was confirmed, we extended laparotomy upwards only if unavoidable under general anesthesia with orotracheal intubation.

In conclusion, AGP remains a common and serious abdominal surgical emergency in resource-poor countries. The outcome depends on several prognostic factors, including length of time before hospitalization, patients' conditions at admission, and management quality. For typhoid ileal perforations, primary repair seams to show better results and lower morbidity. Prevention and proper medical management are fundamental actions to decrease AGP incidence, morbidity, and mortality. Wide availability of experienced and qualified physicians/surgeons certainly would ensure a rapid and effective management of such abdominal surgical emergencies. However, we believe that the presence of general practitioners with adequate surgical skills, even without a specialization, can allow reaching acceptable results in disadvantaged contexts.

## Figures and Tables

**Table 1:** Patients' data.

Variable	Numbers of patients	%
Gender		
Male	38	60.3
Female	25	39.7
Age range (years)		
2–10	17	27.0
10–20	16	25.4
20–30	11	17.4
30–40	8	12.7
40–50	4	6.4
50–60	3	4.7
≥60	4	6.4
Operating time delay range (hours)		
<24	19	30.2
24–48	32	50.8
48–72	5	7.9
Preoperative anemia^*∗*^		
Yes	22	34.9
No	41	65.1
ASA score		
3	47	74.6
4	16	25.4

^*∗*^Hemoglobin <8 g/dL.

**Table 2:** Peritonitis etiology.

	No. of patients	%
Ileal perforation	40	63.5
Gastric-duodenal ulcer perforation	9	14.3
Appendicular	5	7.9
Cryptogenetic	5	7.9
Gynecological	1	1.6
Postoperative	1	1.6
Colonic necrosis by volvulus	1	1.6
Ileal necrosis by hernia strangulation	1	1.6

**Table 3:** Perioperative and postoperative data.

	No. of patients	%
Type of anesthesia		
General anesthesia + orotracheal intubation	33	52.4
Spinal anesthesia + sedation	30	47.6
Main operating procedures		
Simple ileal suture	35	55.6
Antropyloroplasty	9	14.3
Washing drainage	6	9.5
Appendectomy	5	7.9
Ileal resection-ileoileal anastomosis	4	6.3
Wedge ileal resection-ileal suture	2	3.2
Right hemicolectomy-ileocolic anastomosis	1	1.6
Unilateral adnexectomy	1	1.6
Postoperative complications		
Yes	22	34.9
No	41	65.1
Outcome		
Discharge	54	85.7
Deaths	9	14.3

**Table 4:** Distribution of postoperative complications according to Clavien–Dindo.

	Type of complications	*N* (%)
Grade I	Incisional surgical site infection	19 (30.1)
Grade II	Undernutrition requiring parenteral nutrition	7 (11.1)
Grade III	Fecaloid fistula	3 (4.8)
Grade IVa	Acute pulmonary edema	1 (1.6)
Grade IVb	Septic shock	4 (6.3)

**Table 5:** Acute generalized peritonitis etiologies according to sub-Saharan countries.

Authors	Ileal perforation (%)	Gastric or duodenal perforation (%)	Appendicular (%)
Sambo et al. (Benin, 2017) [5]	52.8	17.0	11.3
Kassegne et al. (Togo, 2013) [6]	64.2	16.7	16
Ouangre et al. (Burkina Faso, 2013) [7]	42.5	06.8	33.0
Our series	63.5	14.3	7.9

## Data Availability

The data that support the findings of this study are available from the authors upon request and with permission of Atacora Departmental Hospital Centre, Benin, as restrictions apply to the availability of these data, which were used under license for the current study, and so are not publicly available.

## Abbreviations

CHD-A: Centre Hospitalier Départemental de lʼAtacora
ASA Score: American Society of Anesthesiologists Score
OS: Overall survival
AGP: Acute generalized peritonitis.
